# Spontaneous resolution of chylous ascites following delivery: a case report

**DOI:** 10.1186/1752-1947-6-187

**Published:** 2012-07-04

**Authors:** Inas Babic, Maha Tulbah, Samir Ghourab

**Affiliations:** 1 Department of Obstetrics and Gynecology, King Faisal Specialist Hospital and Research Centre, MBC-52, PO Box 3354, Riyadh 11211, Kingdom of Saudi Arabia

## Abstract

**Introduction:**

Chyloascites or chyloperitoneum, which can be caused by different factors, is a process of eruption of one or many lymphatic vessels spontaneously. Malignant processes, inflammation or trauma can cause a sudden burst in a lymphatic vessel which will lead to a collection of milky fluid in any space of the human body with the abdominal cavity being the most common location. Chyloperitoneum is rare during pregnancy and this case is the fifth described worldwide.

**Case presentation:**

We describe a case of chyloascitis in a 27-year-old primigravida Middle Eastern woman, found coincidentally during cesarean section. Free fluid was found in the abdominal cavity with no source of trauma or masses. An abdominal drain remained *in situ* for six days. The milky fluid was sent for biochemical analysis and found to be positive for triglycerides. Her postoperative course was uneventful. A computed tomography scan of the abdomen and pelvis was negative for fluid collection, tumors or other lesions. While this is the fifth case of chylous ascitis associated with pregnancy, it is the second found to be spontaneous with no obvious cause described to date.

**Conclusion:**

Chylous ascitis is not always associated with tumors, inflammation or trauma. It can, although rarely, be associated with pregnancy. The course of pregnancy is usually uncomplicated in the cases published to date. This fifth case serves as a reminder for obstetricians, when presented with similar findings, to consider chylous ascitis as one of the differential diagnoses. Early diagnosis and appropriate treatment is vital for improved outcomes for the mother and the fetus.

## Introduction

Chylous ascites or chyloperitoneum develops when there is a disruption of the lymphatic system which can occur due to different causes [1]. The most common causes are abdominal malignancies and cirrhosis which account for more than two-thirds of the cases in developed countries, where infectious diseases are prevalent particularly in developing countries. The most common causes in Western countries are abdominal malignancy (especially in adults, in whom lymphoma accounted for at least one third of the cases in one large series of patients identified over 20 years) and cirrhosis (which accounts for over two thirds of all cases). In contrast, infections, such as tuberculosis and filariasis, account for the majority of cases of chylous ascites in Eastern and developing countries. Congenital, inflammatory, postoperative and traumatic causes are less frequent [2,3]. Chylous ascites is a milky, hazy, intra-abdominal fluid rich in triglyceride [4]. It is a rare complication during pregnancy. To the best of our knowledge there have been four cases reported and published. These cases occurred and were recognized during pregnancy or after delivery. Three were found as a complication during pregnancy due to pancreatitis and intestinal volvulus and one was a nontraumatic, coincidental finding during cesarean section [5-8].

## Case presentation

We describe a very similar case of the coincidental finding of chylous ascites during cesarean section. A 27-year-old Middle Eastern primiparous woman was referred from the cardiology unit for antenatal care in our Obstetric High Risk Unit. The patient had multiple comorbidites: morbid obesity with a body mass index (BMI) of 38.9kg/m^2^, congenital restrictive perimembranous ventriculoseptal defect (unrepaired), congenital glaucoma with megalocornea and beta thalassemia trait. There was a positive family history of diabetes, thyroid disease and hypertension, but not hyperlipidemia. She was first seen at 14 weeks of gestation. Ultrasound findings were consistent with gestational age.

A multidiscplinary approach (cardiology, ophtalmology and perinatology) was taken with regular follow up and treatment needed throughout her pregnancy. An echocardiogram showed normal pulmonary pressure, ejection fraction > 55%, and good ventricular function. She was taking: omeprazole 20mg twice daily orally, scopolamine 0.6mg three times daily orally, ferrous sulfate 300mg twice daily orally and timolol (eye drops) one to two drops in each eye daily during pregnancy. Antenatal laboratory investigations showed the following: hemoglobin (Hgb) 108g/L, hematocrit (Hct) 0.337L/L, and a blood group with negative antibodies. Renal and hepatic function tests were within normal limits. Her lipid profile was not indicated. Ultrasound examination at 39 weeks showed evidence of fetal macrosomia and at 40 weeks gestation the decision for a short trial of induction of labor was taken. The presenting part was not engaged in the pelvis (unusual for primigravidas) and the fetus was large for the gestational date. Induction of labor by ripening of the cervix was attempted using 3mg of prostaglandin E2, given in two doses, with no progress. The decision for cesarean section was taken because of the failed induction, and was performed under regional spinal anesthesia. Intraoperatively, upon opening the abdominal cavity, a copious amount of milky fluid was evident. A vigorous baby girl weighing 3720 grams was delivered via a lower segment incision. The uterine incision was closed in two layers. There were no pathological masses or lesions seen on the uterus, ovaries or fallopian tubes. The omentum was inspected and appeared healthy. No other pathology was seen intraoperatively. A culture swab was taken from the fluid and sent to the microbiology laboratory. Peritoneal lavage was carried out using normal saline. A 15 French Jackson-Pratt® abdominal drain was inserted and the abdominal cavity was closed. Estimated blood loss was approximately 600cc.

Postoperatively, the patient was given intravenous antibiotics: the second generation cephalosporin cefoxitine 1 g intravenous once daily, and gentamicin 80mg intravenous q 8 hours, which were administered every eight hours until discharge from the hospital. Her vital signs remained stable. The Jackson-Pratt® drain continuously drained milky discharge. The complete blood count with differential (CBCD) and renal profile on post-operative day one showed: white blood cells (WBC) 9.56 × 10^9^g/L, Hgb 106g/L, platelets 210 × 10^9^g/L, and neutrophils 77.2%. Analysis of the fluid gave the following results: amylase 23u/L, cholesterol 178g/dl, triglyceride 21mg/dl, albumin 20g/L, bilirubin 14mmol/L, lipase 15u/L, and chilomicrons were negative.

Acid-fast bacillus culture was negative. All other cultures sent for bacterial or fungal growth were negative. Wound and urine cultures were negative. Day three post-cesarean section laboratory investigations were as follows: WBC 5.8g/L, Hgb 93g/L and Hct 0.298L/L. Her renal and coagulation profiles were within the normal range. The patient was started on a low-fat diet and had an uneventful recovery. Computed tomography (CT) scans of the chest, abdomen and pelvis were ordered to rule out malignancy or lung lesions. The results were normal with no evidence of fluid collection or any other pathology. The drain was removed six days post operatively. The total amount of fluid collected was 480cc. She was discharged home on day seven post operatively in generally good condition. The patient had three follow up appointments after discharge: at 14 days, 45 days and one year post operatively. Ultrasound of the abdomen and pelvis showed no collection of fluid. She was finally discharged in satisfactory condition and has remained so.

## Discussion

Chylous ascites occurs due to three possible underlying mechanisms: obstruction of lymph flow due to malignancy, exudation of the lymph through the walls of dilated retroperitoneal vessels that lack valves, or acquired thoracic duct obstruction arising from trauma and causing direct leakage of chyle. Malignancy is a common cause, particularly lymphoma which is the most common tumor. Lymphoma accounts for 33% to 50% of cases. Other neoplastic processes, such as breast, pancreatic, colon, renal, ovarian, and prostatic cancers and carcinoid tumors, have also been identified as a cause [9]. Cirrhosis may account for 1% of patients with chyloperitoneum [10]. Infections have also been recognized as a cause: peritoneal tuberculosis, lymphatic filariasis caused by the parasite *Wuchereria bancrofti* and infection with *Mycobacterium avium-intracellulare* in patients with acquired immune deficiency syndrome (AIDS) [11-13].

Due to its very rare association with pregnancy and conditions which might cause chylous ascites, we searched for possible underlying causes in our patient. Pelvic congestion was present due to an overflow and vasodilatation that develop during pregnancy. This occurred as an effect of progesterone and other placental hormones. Pressure of the gravid uterus on the pelvic vessels is a known cause of traumatic rupture. Hypothetically, spontaneous rupture of the lymphatic vessel is another option, which possibly occurred in our patient. During her cesarean section, the place (lymphatic vessel) of leakage could not be found. There were no signs of injury to the omentum, bowel or lymph nodes, with no masses or lesions identified.

## Conclusions

Our case report is the second published in the literature regarding spontaneous chyloperitoneum, without underlying pathology found during pregnancy. It serves as a reminder for obstetricians to keep in mind differential diagnoses of possibile rare associations between two unrelated conditions: chylous ascites and pregnancy. It is vital to emphasize the importance of early diagnosis and identification of the underlying cause where possible, so treatment can be directed to the best care for the future mother and her infant.

## Consent

Written informed consent was obtained from the patient for publication of this case report and any accompanying images. A copy of the written consent is available for review by the Editor-in-Chief of this journal.

## Competing interests

The authors declare that they have no competing interests.

## Authors’ contribution

IB participated in medical management and wrote the case report. MT carried out the antenatal and postnatal management and supervised the coordination and elaboration of the case report. SG participated in medical management and supervised elaboration of the case report. All authors read and approved the final manuscript.
